# Exploring the Relationship between Turkish Mothers’ Parenting and Psychological Well-Being in Early Childhood: The Role of Child Emotion Regulation and Dysregulation

**DOI:** 10.3390/bs14060426

**Published:** 2024-05-21

**Authors:** Ceren Kılıç, Raziye Yüksel Doğan, Emine Nilgün Metin

**Affiliations:** 1 Department of Child Development, Vocational School of Social Sciences, Baskent University, 06790 Ankara, Turkey; 2 Department of Child Development, Faculty of Health Sciences, Hacettepe University, 06100 Ankara, Turkey; raziye.yuksel@hacettepe.edu.tr (R.Y.D.); eminenilgun.metin@hacettepe.edu.tr (E.N.M.)

**Keywords:** early childhood, psychological well-being, emotion regulation, parenting

## Abstract

Although the importance of psychological well-being in early childhood is emphasized, the number of studies conducted with children and adolescents in this field is less than those carried out with adults. The present study aimed to explore the role of child emotion regulation in the relationship between parenting and psychological well-being among a sample of Turkish preschoolers aged 5–6. It further examines the mediating role of emotion regulation. The sample consisted of 416 mothers with children aged 5–6 (M_age_ = 5.38, SD = 0.48; 50% girls-boys). We collected the data using the Multidimensional Assessment of Parenting Scale (MAPS), the Emotion Regulation Checklist (ERC), and the Psychological Well-Being Scale for Children (PWBS-C). The findings revealed significant relationships among parenting practices (positive/negative), the child’s emotion regulation/dysregulation, and the child’s psychological well-being. Moreover, the findings revealed that positive parenting, directly and indirectly, affects children’s psychological well-being through children’s emotion regulation. On the other hand, findings disclosed that negative parenting has both a direct and indirect impact on children’s psychological well-being through children’s emotion regulation. Overall, the study may shed light on a possible process in which positive parenting boosts a child’s emotional regulation and psychological well-being among preschoolers.

## 1. Introduction

In his article, Seligman [1] tells a story about his reaction to his screaming daughter. While he was working in the garden, his five-year-old daughter was throwing weeds in the air. Seligman yelled at his daughter because he was highly goal-oriented and impetuous. His daughter walked away, returned, and said she wanted to talk to him.

“*Dad, do you remember before my fifth birthday? I was a whiner from when I was three until I was five. I whined every day. When I turned five, I decided not to whine anymore. It was the hardest thing I have ever done. Moreover, if I can stop whining, you can stop being cranky (Seligman Excerpt, 2002, pp. 3–4).*”

Seligman had gained a great realization through this experience with his daughter. His daughter made him realize that raising a child is not just about fixing her whining. More than that, he realized that raising children means being aware of their potential and enabling them to develop it by creating appropriate environments and conditions [1].

Seligman’s realization undoubtedly offers an ideal perspective on the supportive, positive parenting practices parents want to establish with their children. This anecdote leads us to the purpose of this study. In this study, we investigated the well-being of preschool children in relation to positive and negative parenting practices and emotion regulation.

### 1.1. Parenting and Psychological Well-Being in Children

Individuals’ physical, spiritual, and social well-being all contribute to their health. Problem-focused psychology approaches focus on the disease model of the individual trying to reduce or reset the discomfort [2]. The field of positive psychology, which is becoming increasingly widespread today, involves identifying and developing interventions that increase well-being. It is concerned with the study of the conditions and processes that contribute to the development and optimal functioning of people and addresses all dimensions of human experiences, such as optimism, kindness, gratitude, self-sacrifice, setting worthwhile goals, and examining ways to create healthy families and institutions [3,4]. The first experiences related to many concepts (optimism, psychological well-being, and self-efficacy) that positive psychology is interested in and emphasizes are usually encountered in childhood, and these experiences can potentially affect lifelong development [5]. Therefore, it is crucial to emphasize research based on positive psychology approaches to child development and conduct more in-depth studies in this field [5,6].

One of the key concepts in positive psychology is well-being, which is considered a complex and multidimensional concept that shows individuals’ perceptions or evaluations of their quality of life by including their satisfaction with life and subjective evaluations of their emotional states [7,8]. The literature on child development has conceptualized in various ways that children’s well-being is a multilayered concept encompassing physical, emotional, and social dimensions. A growing consensus suggests that we should focus on children’s current and future lives, incorporating objective criteria in addition to certain subjective ones [9]. Well-being is a complex, multidimensional, and interdisciplinary concept that incorporates children’s lives and the social contexts in which they live. From a developmental perspective, Moore [10] evaluates children’s well-being and suggests that both individual and environmental factors influence psychological well-being. Moore. [10] addresses well-being in children by encompassing the whole child with four dimensions: physical health, development, and safety; psychological and emotional development; social development and behavior; and cognitive development and educational achievement. From this perspective, psychological well-being includes various positive situations that foster personal development and growth, encompassing cognitive, emotional, and psychological aspects of an individual’s life [11]. Since childhood is recognized as a critical period that shapes an individual’s mental and emotional well-being [12,13], there is an increasing interest in identifying the factors contributing to well-being during this period [14,15,16]. Well-being includes children’s current lives and how the present affects their future and development [17]. Children’s well-being is critical to society’s well-being. Supporting children’s well-being is important for ensuring they have a good childhood and laying a solid foundation for their well-being as future adults [18]. Children’s vital well-being allows them to interact positively and safely with their environment, allowing them to take full advantage of learning opportunities [19]. According to Maslow [20] children who are healthy, happy, and secure are eager to develop and mature. Thus, they enjoy development and progress by acquiring new skills, capacities, and strengths. Even though the importance of psychological well-being in early childhood is emphasized, it seems that the number of studies conducted with children and adolescents in this field is less than the studies performed with adults [21,22,23]. Therefore, recognizing the indicators of well-being in children from the early stages of development is deemed critical to understanding children’s developmental needs and supporting them to lead healthy and happy lives.

In the literature, parenting is a central concept linked to children’s well-being [24,25]. Parents are uniquely essential and irreplaceable persons with whom children form long-term emotional bonds [26], and parents’ attitudes towards their children are pivotal determinants of children’s well-being and mental health [27].

Parenting is conceptualized in various studies using different dimensions. Baumrind [28,29], who conducted the first conceptualizations regarding parenting based on the concept of control, identified three parenting styles: authoritarian, authoritative, and permissive. Authoritarian parents shape their children’s attitudes and behaviors according to certain behavioral standards, demand respect for authority, and value obedience. Authoritative parents value autonomous will and disciplined compliance, approve of the child, and partially limit them. Permissive parents allow the child to regulate their own activities, approve of the child’s desires, and apply very little control. Maccoby and Martin [30], who further developed Baumrind’s [28,29,31] work, considered parenting behaviors in terms of two dimensions, support and control, and identified four parenting behaviors: authoritarian, authoritative, permissive, and neglectful.

Skinner et al. [32] defined the dimensions of positive parenting as parental warmth (open expression of love and care and emotional availability), autonomy support (allowing freedom of expression and action, encouraging the child to value, accept, and value preferences and opinions), and structure (providing information about ways to achieve desired outcomes, predictability, and consistency); and rejection (harsh, overreactive, critical, disapproving, active hatred, disgust, and hostility), coercion (overly restrictive, controlling, authoritarian, and intrusive interactions), and chaos (inconsistent and irregular interactions, and blocking or concealing paths to goals) as negative dimensions of parenting. In Türkiye, it is noteworthy that parenting scales developed and adapted parallel to the trends in Western countries are predominantly shaped within the theoretical framework proposed by Baumrind [28,29] and Maccoby and Martin [30], focusing on the dimensions of authoritative, authoritarian, and neglectful parenting. However, there are relatively few parenting scales for parents of preschool-aged children in Türkiye [33]. One of the challenges encountered in assessing parenting is the limited sensitivity to changes in parenting practices throughout child development. Parenting is a dynamic concept that changes throughout child development [34], and since parenting behaviors incorporate individual, social, and cultural differences [35,36], it is paramount to evaluate parenting from a multifaceted perspective. Schaefer [37], who developed a multidimensional perspective on parenting, proposed a hierarchical model for parenting behaviors, conceptualizing parenting with dimensions of hostility towards affection and control towards autonomy. Parent and Forehand [34], expanding on Schaefer’s [37] work, conceptualized parenting practices within two main bands: comprehensive positive parenting (proactive parenting, positive reinforcement, warmth, and supportiveness) and comprehensive negative parenting (hostility, physical control, and low monitoring). Therefore, this study employs Parent and Forehand’s [34] definition of positive and negative parenting, which employs a multidimensional model to explain parenting behaviors.

In the literature, negative parenting is associated with destructive effects on children’s behavioral and emotional functioning [38], while positive parenting is associated with psychosocial, mental, behavioral, and physical health and well-being outcomes in children [39,40,41]. The importance of positive parenting based on positive psychology has come to the fore [1,42,43]. In the related literature, many interventions support children’s well-being by improving positive parenting and reducing negative parenting [44,45,46]. Positive parenting is linked with many concepts, including reducing emotion regulation difficulties in children [47], well-being [25], psychological resilience [48,49] being a protective factor against emotion regulation and externalization problems [50], and psychological well-being [51]. In light of the relevant literature, although it is reasonable to conclude that psychological well-being in preschool children is significantly predicted by parents’ positive and negative parenting, the mechanisms underlying this relationship still need to be fully elucidated. Therefore, in our study, we considered children’s emotion regulation skills and emotional regulation problems, two mechanisms underlying this relationship.

### 1.2. Emotion Regulation/Dysregulation and Parenting

The relevant literature suggests that children’s emotion regulation also relates to parents’ attitudes toward children. Rohner and Khaleque [26] stated that children’s emotional security and well-being depend on the quality of their relationships with their parents. The development of emotion regulation in young children is mainly determined by the parents’ support [30,52,53]. In this regard, meta-analysis and longitudinal studies show that children exposed to negative parenting face many risks, such as emotion regulation difficulties [47,54], behavioral problems and disruptive behaviors [55,56], inappropriate behaviors [57], depression, and internalizing problems [58]. Restrictive, authoritarian parenting is associated with difficulties in emotion regulation in children [59,60]. Research, on the other hand, shows that positive parenting is associated with more effective emotion regulation in children [61,62] and social–emotional adjustment [63], and positive parenting behaviors increase positive emotions such as joy in children [64,65]. In the family environment, parental warmth, emotional support, and expression of emotions provide many opportunities for children to develop effective emotion regulation skills [66,67]. Therefore, parents’ positive and negative parenting practices were thought to be essential to children’s emotion regulation and dysregulation. 

### 1.3. Emotion Regulation/Dysregulation and Psychological Well-Being

Psychological well-being is also related to emotion regulation, which is becoming increasingly crucial in child development [68]. Emotion regulation refers to how people respond to, manage, and modify their emotional experiences to achieve individual goals and meet environmental demands [53]. More specifically, it is a concept related to our efforts to influence our emotions and how they are expressed [69]. The role of emotion regulation on well-being is underlined in the literature [70,71,72,73]. Emotion regulation is considered a significant indicator of well-being in the literature. Regulating emotions is necessary for adaptive functionality, and inadequate or dysfunctional emotion regulation is associated with poor well-being [69]. Moreover, an increase in the capacity to regulate emotions is linked to healthy mental health and higher levels of subjective well-being. Conversely, difficulties in emotion regulation may lead to various mental health issues and a decrease in happiness and satisfaction in one’s life [74]. Evidence from meta-analyses and longitudinal studies shows that emotion regulation difficulties in children are simultaneously and longitudinally connected with many risks, such as externalization problems [75] and psychopathological indicators [76,77]. Evidence suggests that emotion regulation strategies have a protective function against developmental psychopathology and are associated with well-being, whereas emotion regulation difficulties are a key risk factor for psychopathological symptoms and well-being later in development [77,78,79]. Therefore, it seems reasonable to argue that children’s emotion regulation skills could potentially impact their psychological well-being.

## 2. Current Study

In this study, we aimed to examine the interrelationships between positive and negative parenting of Turkish mothers with preschool children, children’s emotion regulation, regulation problems, and psychological well-being, and to explore whether emotion regulation has a potential role in the relationship between positive and negative parenting and psychological well-being. As far as we know, our study is pioneering in revealing the relationship between positive/negative parenting, children’s emotion regulation, and the psychological well-being of children in early childhood. Relying on the previous research, we held the following hypotheses:

**Hypotheses** **1.**  *Positive/negative parenting, emotion regulation, and lability/negativity are associated with psychological well-being among preschoolers.*

**Hypotheses** **2.**  *Psychological well-being is predicted by positive/negative parenting and emotion regulation in preschoolers.*

**Hypotheses** **3.**  *Emotion regulation and lability/negativity mediate the positive-parenting–psychological well-being relationship in preschoolers.*

**Hypotheses** **4.**  *Emotion regulation and lability/negativity mediate the negative-parenting–psychological well-being relationship in preschoolers.*

## 3. Method

### 3.1. Participants and Procedures

The population of our cross-sectional study consisted of children aged 5–6 years and their Turkish heterosexual mothers. Our study determined the minimum sample size to be 394 with 5% precision and a 95% confidence interval [80]. We collected data to stay within the minimum sample size to avoid the possibility of missing data. 

We obtained approval from the Hacettepe University Ethics Commission (E-35853172-302.08-00002956330) before our research and complied with the ethical standards specified in the 1964 Declaration of Helsinki at every step of our research. We collected the data between 21 July 2023 and 9 December 2023, using an online Google Form that included information about the research and a consent form. We asked participants not to share their personal information to ensure confidentiality and anonymity. We also provided flexibility by allowing participants to stop responding at any stage. We only included participants who voluntarily agreed to participate after being informed. In our study, we used convenience sampling to reach as many participants as possible, both economically and practically. We asked mothers with more than one child to answer the questions by considering only one of their children between the ages of 5 and 6. We collected data from 437 mothers in our study, but we excluded the data of 21 mothers with outliers from the analyses due to concerns about cleaner data. Demographic characteristics of the participants are presented in Table 1.

As shown in Table 1, 416 Turkish heterosexual mothers with at least one child aged 5–6 years were included in the study. The average age of the children was 5.38 ± 0.48 years. Of the mothers, 69.71% were between the ages of 31 and 40, 81.97% had a university education, and 60.82% were employed. Of the fathers, 64.66% were between the ages of 31 and 40, 75.49% had a university degree, and 98.02% were employed.

### 3.2. Measures

#### 3.2.1. Descriptive Characteristics Questionnaire

We collected the descriptive characteristics of the participants by creating a demographic information form. With this form, we asked the participants to report the age of parents and children, gender of children, parental education level and employment status, and family structure.

#### 3.2.2. Multidimensional Assessment of Parenting Scale

The Multidimensional Assessment of Parenting Scale (MAPS) was developed by Parent and Forehand [34]. The scale aims to assess parents’ parenting styles based on self-report. The MAPS consists of seven subscales: proactive parenting, positive reinforcement, intimate relationship, supportive approach, hostility, low control, and physical control. The sum of MAPS, hostility, low control, and physical control sub-dimensions constitute the broadband negative parenting scale. In contrast, the sum of proactive parenting, positive reinforcement, supportive approach, and intimate relationship sub-dimensions constitute the broadband positive parenting scale (e.g., I use threats as punishment with little or no justification; I express affection by hugging, kissing, and holding my child.). The adaptation of MAPS into Turkish was conducted by Karababa [81]. The MAPS consists of 34 items, and responses are scored on a Likert-type scale ranging from “1 (never)” to “5 (always)”. High scores on the scale for the broadband positive parenting dimension indicate a highly positive parenting whereas high scores on the scale for the broadband negative parenting dimension indicate a highly negative parenting. In the present sample, confirmatory factor analysis (CFA) results supported an acceptable fit between the model and the data, as indicated by the following indices: S-Bχ^2^ = 1054.978, *p* < 0.001; GFI = 0.93; CFI = 0.96; TLI = 0.96; SRMR = 0.06; RMSEA = 0.03 (90% CI: 0.03–0.04). Cronbach’s alpha internal consistency coefficient was 0.802 for the broadband negative parenting subscale and 0.779 for the broadband positive parenting subscale of MAPS. 

#### 3.2.3. Emotion Regulation Checklist

The Emotion Regulation Checklist (ERC) was developed by Shields and Cicchetti [82]. The checklist aims to assess children’s emotion regulation skills. The ERC consists of two sub-dimensions: lability/negativity and emotion regulation. The adaptation of the ERC into Turkish was conducted by Batum and Yagmurlu [83]. The ERC consists of 24 items, and responses are scored on a Likert-type scale ranging from “1 (Never/rarely)” to “4 (Almost always)” (e.g., responds positively to friendly or neutral approaches of peers; prone to outbursts of anger, tantrums). High scores on the scale for the lability/negativity subscale indicate greater regulation problems. In the present sample, the results of the confirmatory factor analysis (CFA) support the model fit indices discussed above, which generally indicate a satisfactory-to-good fit between the model and the data: S-Bχ^2^ = 917.565, *p* < 0.001; GFI = 0.93; CFI = 0.91; TLI = 0.90; SRMR = 0.08; RMSEA = 0.06 (90% CI: 0.06–0.07). Cronbach’s alpha internal consistency coefficient was 0.679 for the emotion regulation subscale and 0.789 for the lability/negativity subscale. 

#### 3.2.4. Psychological Well-Being Scale for Children

The Psychological Well-Being Scale for Children (PWBS-C) was developed by Atan and Buluş [84]. The scale aims to measure children’s psychological well-being based on parents’ responses to questions about the frequency of various behaviors in children aged 5–6. The PWBS-C consists of 66 items and 9 sub-dimensions: physical health, low internalizing, low externalizing, self-regulation, social competence–assertiveness, cognitive competence, value behaviors, psychological resilience, and life satisfaction; responses are scored on a Likert-type scale ranging from “1 (Never)” to “5 (Always)”. High scores on the scale indicate a high level of psychological well-being. In the present sample, confirmatory factor analysis (CFA) results supported an acceptable fit between the model and the data, as indicated by the following indices: S-Bχ^2^ = 4766.571, *p* < 0.001; GFI = 0.93; CFI = 0.97; TLI = 0.97; SRMR = 0.07; RMSEA = 0.03 (90% CI: 0.03–0.04). We calculated the Cronbach’s alpha internal consistency coefficient for the psychological well-being scale as 0.936 in the current sample.

### 3.3. Data Analysis

Data were analyzed using an open-source analysis program Jamovi 2.4.11, JASP 0.18.3.0 and SPSS v25. Before starting the analyses, the characteristics of the observed scales were analyzed, and the assumptions of the analyses were checked. The normality assumption was interpreted through skewness and kurtosis coefficients [85]. The z-standard score was calculated for each variable to determine univariate outliers. Accordingly, 14 participants with z-standard scores, except for −3 and +3 [86] were excluded from the analyses. Mahalanobis values were calculated to detect multivariate outliers. To this end, 2 participants with a value less than 0.001 were excluded from the analysis. Afterward, a thorough analysis of the validity and reliability of the scales used in our sample was conducted. The analysis of Mardia’s multivariate skewness and kurtosis values indicated that the scales did not satisfy the assumption of multivariate normality, necessitating the use of diagonally weighted least squares (DWLS) in this research, particularly for the analysis of Likert-type measurement instruments [87]. Confirmatory factor analysis (CFA) and several fit index values were also performed, including GFI, CFI, TLI, SRMR, and RMSEA. The fit index values must be CFI > 0.90 [88], GFI > 0.90 [88,89] TLI > 0.90 [88], SRMR < 0.08 [88], and RMSEA < 0.08 [89] for them to be considered reasonable. The scale’s reliability was examined using Cronbach’s alpha internal consistency coefficients. Moreover, since the data in the study were collected through self-report measures, the presence of common method bias was investigated using Harman’s single-factor test [90,91]. The relationships between the measurements were examined with the Pearson product–moment correlation coefficient. Then, by checking the regression analysis assumptions, whether the effect of an independent variable (positive/negative parenting) on a dependent variable (psychological well-being) was mediated by a third variable (emotion regulation/dysregulation) was tested with a mediation model. The effect of indirect effects was assessed using the bootstrap method with 95% confidence intervals and 5000 resampling [92]. 

## 4. Results

### 4.1. Common Method Bias

Since the data in this study were collected through self-report measures, the presence of common method bias was examined using Harman’s single-factor test [90,91]. Accordingly, all items in the Multidimensional Assessment of Parenting Scale, Emotion Regulation Checklist, and Psychological Well-Being Scale for Children were subjected to exploratory factor analysis (EFA). According to the results, there were 32 factors with eigenvalues greater than 1, with the initial value of the first factor being 19.71, explaining 15.26% of the variance. This is less than the critical value of 40%. Therefore, it was concluded that there was no common method bias in the study [90,91].

### 4.2. Preliminary Analyses

Means, standard deviations, and correlations for continuous variables in the study are presented in Table 2.

As illustrated in Table 2, we found significant relationships between variables. Findings show that positive parenting has a positive relationship with the child’s emotion regulation (r = 0.29, *p* < 0.01) and psychological well-being (r = 0.28, *p* < 0.01), and that there is a significant negative relationship with the child’s regulation problems (r = −0.12, *p* < 0.05). On the contrary, negative parenting was negatively related to the child’s emotion regulation (r = −0.193, *p* < 0.01) and psychological well-being (r = −0.39, *p* < 0.01), and there was a positive relationship with the child’s regulation problems (r = 0.43, *p* < 0.01). Additionally, there was a positive relationship between psychological well-being and emotion regulation (r = 0.67, *p* < 0.01), and a significant negative relationship was detected with the regulation problem (r = −0.62, *p* < 0.01). As can be seen, there is no multicollinearity (r > 0.90) [85].

### 4.3. The Results of Regression and Mediating Analyses

First, the role of emotion regulation and regulation problems in the relationship between positive parenting and children’s psychological well-being was scrutinized. To that end, a multiple mediator analysis revealed that positive parenting significantly influenced children’s emotion regulation, regulation problems, and psychological well-being. In addition, as can be seen in Table 3, children’s psychological well-being is significantly predicted by emotion regulation and regulation problems. 

**Figure 1:**
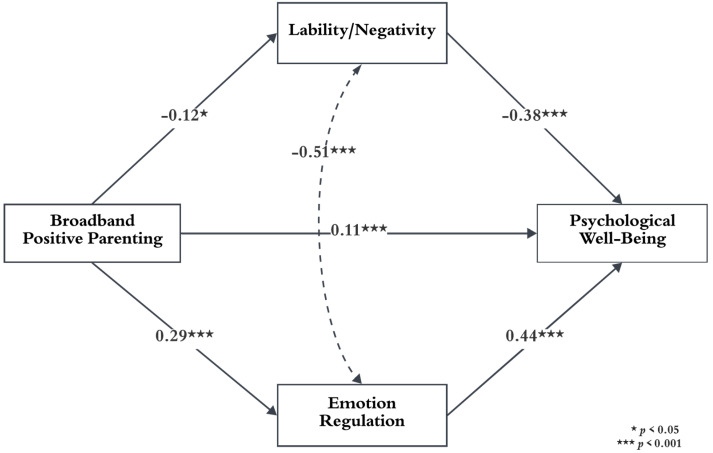
Conceptual framework of the study and the mediation role of lability/negativity and emotion regulation in the relationship between positive parenting and psychological well-being.

Table 4 demonstrates the partial mediating role of emotion regulation and regulation problems in the relationship between positive parenting and psychological well-being. The findings showed that positive parenting directly and indirectly influences psychological well-being through emotion regulation. Accordingly, 61.07% of the total effect was indirect. While 45.19% of the indirect effect is the role of emotion regulation in the relationship between positive parenting and psychological well-being, 16.12% is the role of regulation problems. These results suggest that emotion regulation is relatively more important in the relationship between positive parenting and psychological well-being. In line with these results, mothers’ increased positive parenting enhances children’s psychological well-being by increasing emotion regulation and decreasing regulation problems. Standardized regression estimates for the proposed model are presented in Figure 1. 

Following the results, the role of emotion regulation and regulation problems in the relationship between negative parenting and children’s psychological well-being was investigated. The multiple mediator analysis revealed a significant predictive effect of negative parenting on children’s emotion regulation, regulation problems, and psychological well-being. As seen in Table 5, children’s psychological well-being is significantly predicted by emotion regulation and regulation problems. 

Table 6 depicts the partial mediating role of emotion regulation and dysregulation in the relationship between negative parenting and psychological well-being. The findings unveiled that negative parenting, directly and indirectly, impacts psychological well-being through emotion regulation. More specifically, 57.57% of the total effect is indirect. While regulation problems account for 33.43% of the indirect effect in the relationship between negative parenting and psychological well-being, emotion regulation accounts for 24.14%.

**Figure 2:**
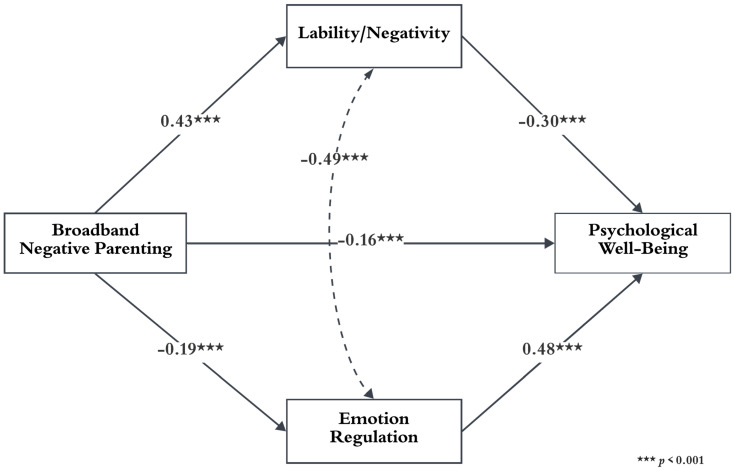
Conceptual framework of the study and the mediation role of the lability/negativity and emotion regulation in the relationship between negative parenting and psychological well-being.

These results uncover that regulation problems are relatively more important in the relationship between negative parenting and psychological well-being. In line with these results, mothers’ increasing negative parenting decreases children’s psychological well-being by both increasing regulation problems and decreasing emotion regulation. Figure 2 displays the standardized regression estimates for the proposed model.

## 5. Discussion

This study presents insights into the relationship between Turkish mothers’ parenting and the psychological well-being of 5–6-year-old children in Ankara, Türkiye. To explore this dynamic, an elaborate path model was devised, considering the mediating role of children’s emotion regulation and dysregulation. Despite the strong emphasis on the central role of parenting in children’s healthy growth and development [12,13], it is remarkable that their role in supporting psychological well-being, especially in the preschool period, needs a more in-depth focus. In this respect, given that the starting age for primary school in our country is 5–6 years and the importance of early functionality for later success and well-being, our study focused on mothers of children aged 5–6. As far as we know, our study is pioneering in revealing the relationship between positive and negative parenting, children’s emotion regulation, and psychological well-being in early childhood. 

Our findings have shown, unsurprisingly, that children’s psychological well-being is associated with their mothers’ positive and negative parenting practices. Accordingly, as mothers’ positive parenting practices increase, children’s well-being also increases, whereas as negative parenting practices increase, children’s well-being decreases. Our findings suggest the potential of positive parenting to support children’s psychological well-being in early childhood. In a similar vein, the negative correlation between negative parenting and children’s well-being implies that mothers’ negative parenting practices may have adverse effects on children’s psychological well-being. Consistent with our study, when examining the relevant literature, it has been seen that both positive parenting [24,40,93,94] and negative parenting [95,96] are associated with children’s levels of psychological well-being. Furthermore, in line with previous research, our research revealed that children’s psychological well-being was significantly predicted by their emotional regulation and dysregulation. These results echo previous research [97,98,99]. These results suggest to us that the effective regulation of emotions by children aged 5–6 plays a significant role in enhancing their overall psychological well-being. 

Based on our research findings, positive parenting directly and indirectly affects children’s psychological well-being by regulating their emotions. The direct effect of positive parenting is its positive impact on children’s psychological well-being. On the other hand, the indirect effect suggests that this relationship may be partially realized through regulating children’s emotions and dysregulation. Considering that parents have a unique role in children’s lives [26], it seems reasonable to think that an essential indicator of children’s psychological well-being would be related to parents’ positive and supportive parenting approach [24,40,93,94]. The indirect effects found in our study are important in highlighting the critical role of children’s emotion regulation skills in the relationship between positive parenting and psychological well-being. Our results indicate that positive parenting not only directly influences children’s psychological well-being but also contributes to their ability to effectively manage their emotions, which in turn enhances psychological well-being. Resonating with prior research, the results show that positive parenting positively affects the child’s emotion regulation skills [100,101], and the results also showed that there was a significant negative relationship with the child’s emotion dysregulation [102,103]. These results align with many studies reporting that positive parenting has an important role in increasing children’s ability to regulate their emotions and reducing regulation problems [104,105,106]. Seligman [1] emphasizes the importance of positive parenting practices that increase children’s positive emotions up to 6 years of age and include frequent and unconditional modeling of positive emotions. A recent study points out that parents’ positive parenting practices can create a solid foundation for children to successfully regulate their emotional state [107]. As already demonstrated in previous studies, positive parenting involves aspects such as supportiveness, emotional accessibility, clear expressions of affection, fostering intimate relationships, and providing children with a predictable and consistent parenting style [32,34]. Moreover, non-coercive control and authoritative parenting practices create an environment where children perceive their thoughts and ideas as valued and accepted [108]. From another perspective, positive parenting is associated with concepts such as acknowledging the child’s emotions, discussing emotions with the child, helping the child verbally express their emotions, discussing situations that evoke emotions, and having coping skills for these situations [109,110]. Indeed, research indicates that approaches aimed at teaching problem-solving and emotion regulation to children are positively associated with positive parenting practices like authoritative parenting while negatively associated with negative parenting practices like authoritarian approaches that disregard emotions [111]. This result is also consistent with theoretical models that suggest that young children develop their regulation skills in the context of mother–child interactions by observing their mothers’ supportive approaches [112]. Furthermore, mothers valuing children’s emotions and not belittling their feelings predict less emotional lability in children [113]. These findings suggest that positive parenting practices, where children feel unconditionally accepted and are given the opportunity to express both positive and negative emotions openly, create an appropriate environment for children to learn how to regulate their emotions. This result also implies an important consideration for emotion-focused approaches in clinical and educational interventions related to positive parenting. 

Our research also shows that negative parenting has both a direct and indirect impact on children’s psychological well-being through emotion regulation and dysregulation. While the direct effect represents the negative impact of negative parenting on psychological well-being, the indirect effect implies that this relationship may be partially realized through emotion regulation and dysregulation. Extensive research has revealed that children exposed to negative parenting face a multitude of risks that can significantly impact their overall well-being [95]. As previously mentioned, negative parenting has been associated with destructive effects on children’s behavioral and emotional functioning [38] and social problems [95]. Additionally, children exposed to negative parenting may develop behavioral problems, disruptive behaviors [55,56], and inappropriate behaviors [57], childhood anxiety [114], and, furthermore, depression and internalization/externalization problems [58,95]. These risks also include difficulties in regulating emotions [47,54]. In our study, the indirect effects underscore the critical role of emotion regulation and emotion dysregulation in the relationship between negative parenting and psychological well-being. Our results indicate that negative parenting not only reduces children’s psychological well-being but also has the potential to affect children’s emotion regulation skills. Furthermore, children’s difficulties in emotion regulation have the potential to negatively impact their psychological well-being. Congruent with previous studies, our results suggest that negative parenting negatively affects the child’s emotion regulation skills [62]. Considering that the adult’s emotional response is one of the variables with the greatest impact on the child’s emotion regulation [15], it is more reasonable to expect that children exposed to negative parenting practices, which involve emotional detachment, rejection, and excessively restrictive behaviors [32,33,34], would experience difficulties in emotion regulation. It can be considered a natural consequence that increased difficulties in emotion regulation negatively impact children’s well-being. Hence, the literature shows that unsupportive parenting practices increase emotion regulation difficulties in children [47,54,115,116] and that emotion regulation difficulties reduce psychological well-being [117,118]. Our findings, deriving from both models, contribute to the literature by emphasizing the critical role of mothers’ positive parenting approaches in enhancing both psychological well-being and the development of emotion regulation skills in their children during early childhood.

Parenting, which involves caregiving, successful adaptation of children, and views on child development, varies across different cultures [35,36,119,120]. Therefore, we can consider our research findings in a cultural context and draw inferences for future studies. Researchers have identified cultures that prioritize values such as independence, autonomy, self-confidence, individualism, and personal achievement as individualistic, while cultures that emphasize values such as group cohesion and goals, loyalty, and interdependence are known as collectivistic [121]. Additionally, there are cultures that embody both individualistic and collectivistic characteristics, known as autonomous relational self [122]. However, individualism and collectivism can coexist in a given culture [120]. Studies on the cultural dimension of parenting generally show that parenting practices in individualistic cultures involve values such as democracy, support, equality, independence, and individuality of the child [123]. Meanwhile, in collectivistic cultures, child-rearing involves conforming to the group to which one belongs, hierarchy, and obedience [120,124]. In cultures where autonomous relational self is adopted, children are raised under significant control in an atmosphere where there is mutual emotional attachment and loyalty between children and family members, and autonomy is also emphasized [124]. Furthermore, Kağıtçıbaşı [122,124] introduced three distinct family models according to the family change theory: family model of independence, family model of interdependence, and family model of psychological interdependence. In the dependence family model, there is interdependence and hierarchy, and child-rearing is oriented towards obedience. The independence family model values the child’s autonomy and self-confidence. In the psychological interdependence family model, which is a synthesis of the two models, autonomy is valued in parenting. However, emotional attachments still exist. Since Türkiye is a country that is influenced by the cultural characteristics of both the East and the West due to its geographical location, the autonomous relational family structure is widely seen in Türkiye [122]; in this context, in our study on mothers in the Turkish population, it is seen that mothers include parenting practices related to both individualistic and collectivist cultures in their understanding of parenting as a part of autonomous relational culture. This may have been effective in Turkish mothers’ reflection of positive and negative parenting practices in the child-rearing process in the current study.

### Limitations, Implications and Future Directions

Our research is subject to certain limitations. Firstly, although Türkiye is a country that harbors diverse ethnic backgrounds and receives immigrants from various countries (such as Syria, etc.), this study only included mothers who are literate Turkish citizens living in Türkiye. The primary reason for this limitation is that the scales used in the research have been adapted to the Turkish language. Therefore, it is recommended that future studies adapt the scales for other ethnic backgrounds and test the model with parents of diverse ethnicities. Culture-specific research will expand our understanding of parenting practices. Moreover, most studies refer only to mothers, and generalizations to fathers are limited. Secondly, measurements based on mothers’ self-parts were used in the study. This may have elicited socially desirable responses, which had the potential to obscure the internal boundary. However, in our study, we could not receive direct feedback from children due to the lack of psychological well-being measures adapted or developed into Turkish for the 5–6 age range. Therefore, it is recommended that tools measuring children’s self-reported psychological well-being be developed in future studies. Thirdly, another significant limitation of our study is the issue of heteronormativity. This problem still persists as a societal stigma, particularly in Türkiye. Therefore, more research is needed to reflect the ideologies of non-heterosexual mothers. Fourth, a cross-sectional design was used in our research, so it is impossible to discuss causality between the variables. Longitudinal or experimental designs can be designed to test potential mediation models in the relationships between these parenting practices and children’s psychological well-being. Fifth, the proposed model may have confounding variables (mothers’ emotion regulation difficulties or stress, family relationships and communication patterns, co-parenting and fathers’ parenting approaches, etc.). Sixth, our sample included mothers with preschool children aged 5–6 in Ankara, Türkiye, so further research is required to increase the generalizability of our findings. Despite these limitations, our study is critical because it underlines the role of emotion regulation and regulation problems in the relationship between mothers’ parenting and the psychological well-being of 5–6-year-old children. 

As stated before, well-being includes children’s current lives and how the present affects their future and development [17], and children’s psychological well-being influences society’s well-being [18]. Additionally, it is possible for children, especially in early childhood, to develop emotion regulation skills in a family where parents accept emotions and effective emotion regulation strategies are modeled [98,125,126]. At this point, we predict that positive parenting that includes supportive, warm, emotion-focused approaches can significantly contribute to psychological well-being by increasing emotional regulation in children. Therefore, we believe that the results of our research can shed light on future researchers and educators. Intervention programs aimed at improving parenting practices can provide additional training for children to develop conscious and responsible behaviors for emotional regulation. Future research initiatives could extend the existing model by examining the impact of co-parenting and the father’s role in the current model. On the other hand, further research can examine the relationship between positive and negative parenting and children’s psychological well-being in different periods of development or different cultures, evaluate it from a longitudinal perspective, develop positive parenting intervention programs, and reveal the indicators of this intervention program for children. These studies may help us understand this relationship from a more holistic perspective.

## 6. Conclusions

In this study, we examine the relationships between positive and negative parenting of Turkish mothers with preschool children, children’s emotion regulation and dysregulation, and psychological well-being to explore whether emotion regulation has a potential role in the relationship between positive and negative parenting and psychological well-being. In other words, this study documented the relationship between the positive and negative parenting of mothers with children aged 5–6 and their children’s psychological well-being, and the partial mediator role of children’s emotion regulation skills and regulation problems in this relationship. Our research has the potential to fill an essential gap in the literature by addressing the role of child emotion regulation and dysregulation in the relationship between the positive and negative parenting of mothers of 5–6-year-old preschool children and the children’s psychological well-being. The results revealed the importance of mothers’ parenting by emphasizing the potential impact of parents’ behaviors on children’s psychological well-being in the early years. Our findings also highlight the need to promote positive parenting and reduce negative parenting. These findings have significant implications for parents, educators, and mental health professionals who work with children and their families.

## Figures and Tables

**Table 1:** The demographic characteristics of the participants (n = 416).

Variables	n (%) or M ± SD
Age of children (5–6 years)	5.38 ± 0.48
Gender of children	
Female	208 (50.00)
Male	208 (50.00)
Number of children in families	
Single child	194 (46.63)
Two children	187 (44.95)
More than two children	35 (8.41)
Family structure	
Nuclear family	362 (87.02)
Extended family (parent/child/grandparent)	37 (8.89)
Single parent family	17 (4.08)
Age categories of mothers	
30 and under 30	71 (17.07)
Between 31 and 40	290 (69.71)
41 years and above	55 (13.22)
Education levels of mothers	
Elementary	21 (5.05)
Highschool	54 (12.98)
University	341 (81.97)
Associate degree	52 (12.50)
Bachelor degree	212 (50.96)
Postgraduate	77 (18.51)
Employment status of mothers	
Not employed	163 (39.18)
Employed	253 (60.82)
Age categories of fathers	
30 and under 30	27 (6.49)
Between 31 and 40	269 (64.66)
41 years and above	120 (28.85)
Education levels of fathers	
Elementary	13 (3.12)
Highschool	89 (21.39)
University	314 (75.49)
Associate degree	39 (9.38)
Bachelor degree	196 (47.12)
Postgraduate	79 (18.99)
Employment status of fathers	
Not employed	8 (1.92)
Employed	408 (98.08)
Total	416 (100)

**Table 2:** Descriptive statistics and correlations of the variables.

Variables	Descriptive Statistics						Correlation Coefficients (*r*)				
	Min.	Max.	M	SD	Skewness	Kurtosis	1	2	3	4	5
1. Positive Parenting	53.00	80.00	71.68	5.34	−0.69	0.26	1	−0.23 **	0.29 **	−0.12 *	0.28 **
2. Negative Parenting	19.00	57.00	33.19	7.22	0.65	0.31		1	−0.19 **	0.43 **	−0.39 **
3. Emotion Regulation	18.00	32.00	26.52	3.25	−0.35	−0.63			1	−0.52 **	0.67 **
4. Lability/Negativity	16.00	46.00	27.83	5.92	0.60	0.19				1	−0.62 **
5. Psychological Well-Being	187.00	312.00	260.05	24.48	−0.32	−0.29					1

Note. * Correlation is significant at the 0.05 level (2-tailed). ** Correlation is significant at the 0.01 level (2-tailed).

**Table 3:** Unstandardized coefficients for the mediating role of lability/negativity and emotion regulation in the relationship between positive parenting and psychological well-being.

Antecedent	Consequent			
	M_1_ (Emotion Regulation)			
	*Est.*	*SE*	*t*	*p*
X (Broadband Positive Parenting)	0.177	0.0286	6.20	<0.001
	R^2^ = 0.0849			
	F = 38.4; *p* < 0.001			
	M_2_ (Lability/Negativity)			
X (Broadband Positive Parenting)	−0.133	0.0540	−2.46	0.014
	R^2^ = 0.0145			
	F = 6.07; *p* = 0.014			
	Y (Psychological Well-Being)			
X (Broadband Positive Parenting)	0.507	0.157	3.23	0.001
M_1_ (Emotion Regulation)	3.323	0.299	11.12	<0.001
M_2_ (Lability/Negativity)	−1.555	0.158	−9.83	<0.001
	R^2^ = 0.558			
	F = 173; *p* < 0.001			

SE = standard error; Est. = estimate; X = independent variable; M = mediator variable; Y = outcome or dependent variable.

**Table 4:** Standardized indirect effects of lability/negativity and emotion regulation in the relationship between positive parenting and psychological well-being.

Type	Path	β	SE	BootLLCI	BootULCI	*p*
Indirect	Broadband Positive Parenting ⇒ Emotion Regulation ⇒ Psychological Well-Being	0.1286	0.1085	0.3675	0.8214	<0.001
	Broadband Positive Parenting ⇒ Lability/Negativity ⇒ Psychological Well-Being	0.0452	0.0864	0.0161	0.4124	0.017
Direct	Broadband Positive Parenting ⇒ Psychological Well-Being	0.1107	0.1562	0.2114	0.7960	0.001
Total	Broadband Positive Parenting ⇒ Psychological Well-Being	0.2846	0.2156	0.8202	1.7540	<0.001

Note. Number of bootstrap samples for percentile bootstrap confidence intervals: 5000.

**Table 5:** Unstandardized coefficients for the mediating role of lability/negativity and emotion regulation in the relationship between negative parenting and psychological well-being.

Antecedent	Consequent			
	M_1_ (Emotion Regulation)			
	*Est.*	*SE*	*t*	*p*
X (Broadband Negative Parenting)	−0.0868	0.0217	−4.00	<0.001
	R^2^ = 0.0372			
	F = 16.0; *p* < 0.001			
	M_2_ (Lability/Negativity)			
X (Broadband Negative Parenting)	0.356	0.0363	9.81	<0.001
	R^2^ = 0.189			
	F = 96.2; *p* < 0.001			
	Y (Psychological Well-Being)			
X (Broadband Negative Parenting)	−0.555	0.122	−4.55	<0.001
M_1_ (Emotion Regulation)	3.636	0.285	12.77	<0.001
M_2_ (Lability/Negativity)	−1.228	0.170	−7.21	<0.001
	R^2^ = 0.568			
	F = 181; *p* < 0.001			

SE = standard error; Est. = estimate; X = independent variable; M = mediator variable; Y = outcome or dependent variable.

**Table 6:** Standardized indirect effects of lability/negativity and emotion regulation in the relationship between negative parenting and psychological well-being.

Type	Path	β	SE	BootLLCI	BootULCI	*p*
Indirect	Broadband Negative Parenting ⇒ Emotion Regulation ⇒ Psychological Well-Being	−0.0931	0.0825	−0.502	−0.1426	<0.001
	Broadband Negative Parenting ⇒ Lability/Negativity ⇒ Psychological Well-Being	−0.1289	0.0749	−0.601	−0.2909	<0.001
Direct	Broadband Negative Parenting ⇒ Psychological Well-Being	−0.1636	0.1213	−0.773	−0.3433	<0.001
Total	Broadband Negative Parenting ⇒ Psychological Well-Being	−0.3856	0.1535	−1.616	−0.9907	<0.001

Note. Number of bootstrap samples for percentile bootstrap confidence intervals: 5000.

## Data Availability

The datasets generated and/or analyzed in the study are not publicly available but are only available from the corresponding author upon a reasonable request.
